# Targeted drug delivery into glial scar using CAQK peptide in a mouse model of multiple sclerosis

**DOI:** 10.1093/braincomms/fcad325

**Published:** 2023-11-27

**Authors:** Leila Zare, Safoura Rezaei, Elaheh Esmaeili, Khosro Khajeh, Mohammad Javan

**Affiliations:** Department of Physiology, Faculty of Medical Sciences, Tarbiat Modares University, P.O. Box 14115-331, Tehran, Iran; Institute for Brain and Cognition, Tarbiat Modares University, P.O. Box 14115-331, Tehran, Iran; Department of Nanobiotechnology, Faculty of Biological Sciences, Tarbiat Modares University, P.O. Box 14115-154, Tehran, Iran; Institute for Brain and Cognition, Tarbiat Modares University, P.O. Box 14115-331, Tehran, Iran; Department of Nanobiotechnology, Faculty of Biological Sciences, Tarbiat Modares University, P.O. Box 14115-154, Tehran, Iran; Department of Biochemistry, Faculty of Biological Sciences, Tarbiat Modares University, P.O. Box 14115-154, Tehran, Iran; Department of Physiology, Faculty of Medical Sciences, Tarbiat Modares University, P.O. Box 14115-331, Tehran, Iran; Institute for Brain and Cognition, Tarbiat Modares University, P.O. Box 14115-331, Tehran, Iran; International Collaboration on Repair Discoveries (ICORD), University of British Columbia, Vancouver V6T1Z4, British Columbia, Canada

**Keywords:** multiple sclerosis, extracellular matrix, porous silicon, targeting peptide, methylprednisolone

## Abstract

In multiple sclerosis, lesions are formed in various areas of the CNS, which are characterized by reactive gliosis, immune cell infiltration, extracellular matrix changes and demyelination. CAQK peptide (peptide sequence: cysteine–alanine–glutamine–lysine) was previously introduced as a targeting peptide for the injured site of the brain. In the present study, we aimed to develop a multifunctional system using nanoparticles coated by CAQK peptide, to target the demyelinated lesions in animal model of multiple sclerosis. We investigated the binding of fluorescein amidite–labelled CAQK and fluorescein amidite–labelled CGGK (as control) on mouse brain sections. Then, the porous silicon nanoparticles were synthesized and coupled with fluorescein amidite–labelled CAQK. Five days after lysolecithin-induced demyelination, male mice were intravenously injected with methylprednisolone-loaded porous silicon nanoparticles conjugated to CAQK or the same amount of free methylprednisolone. Our results showed that fluorescein amidite–labelled CAQK recognizes demyelinated lesions in brain sections of animal brains injected with lysolecithin. In addition, intravenous application of methylprednisolone-loaded nanoparticle porous silicon conjugated to CAQK at a single dose of 0.24 mg reduced the levels of microglial activation and astrocyte reactivation in the lesions of mouse corpus callosum after 24 and 48 h. No significant effect was observed following the injection of the same dose of free methylprednisolone. CAQK seems a potential targeting peptide for delivering drugs or other biologically active chemicals/reagents to the CNS of patients with multiple sclerosis. Low-dose methylprednisolone in this targeted drug delivery system showed significant beneficial effect.

## Teaser

Zare *et al*. report that cysteine–alanine–glutamine–lysine peptide efficiently binds to the demyelinated area in sections obtained from animal model of multiple sclerosis. When attached to the surface of a nanoparticle loaded with methylprednisolone, this peptide delivered them to the lesion site following systemic injections to mice with a focal demyelination in the brains.

## Graphical Abstract

**Graphical Abstract:**
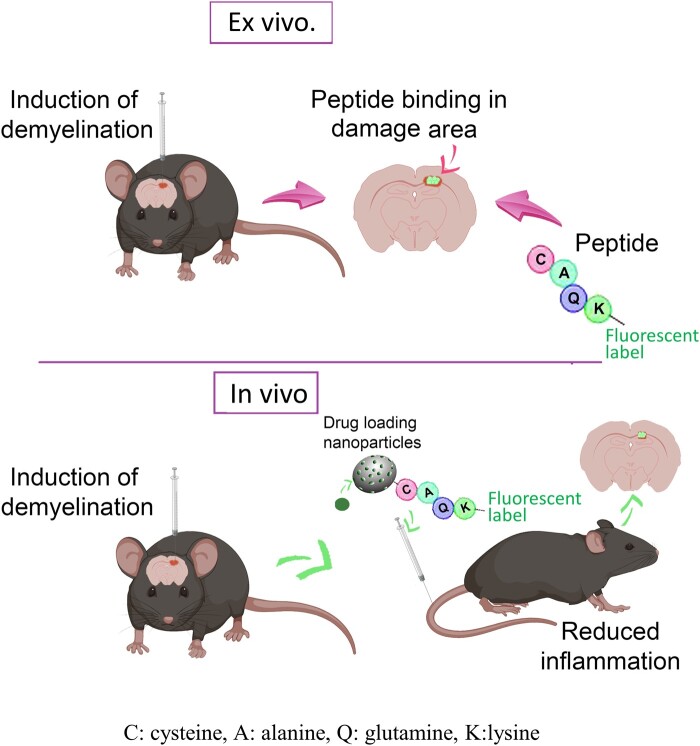

## Introduction

Multiple sclerosis is a neurodegenerative and inflammatory disease associated with astrocytes and microglial activation. Inflammation and consequent tissue damage activate resident macrophages and recruit peripheral immune cells to increase the production of pro-inflammatory cytokines and other inflammatory mediators.^1^ Reactive astrocytes and microglia generate a glial scar that restricts the spread of cytotoxic inflammatory mediators to surrounding areas.^2^ However, in the chronic phase, glial scar inhibits remyelination and axonal regrowth.^3^ Furthermore, the extracellular matrix (ECM) of the CNS is composed of many components with inhibitory effects on regeneration and repair. The ECM is a complex network comprised primarily of glycoproteins, collagens and glycosaminoglycan.^4^

Following CNS insults the composition of many ECM components change due to the alteration of glial cell gene expression and *de novo* production of ECM molecules.^5^ For instance, in the context of multiple sclerosis, the expression of the chondroitin sulphate proteoglycan members including versican, aggrecan and neurocan was changed over the progress of the disease, and their deposition is obvious on the border of chronic multiple sclerosis lesions.^6^ Therefore, reactive astrocytes, inflammatory cells and ECM components may act as important cues for targeted therapies in multiple sclerosis and other neurodegenerative disorders of CNS.^7^

Currently, anti-inflammatory drugs are used for inhibiting and preventing the inflammatory progressive phase of diseases, which are often administered systemically at high doses and lead to significant side effects and drug toxicity.^8^ In this regard, methylprednisolone (MP), a synthetic corticosteroid, is often used for treating clinically significant relapses in multiple sclerosis patients.^9^

Administration of MP is accompanied by various unwanted effects such as hyperglycaemia, increasing the risk of respiratory infections, etc.^10^ Hence, developing safe and effective targeted delivery systems is crucial.^11^

In recent years, there has been much interest in developing novel drug delivery systems, which effectively direct the exact amount of drug to a specific site. This approach reduces the side effects compared with the traditional delivery systems, which diffuse the drug across the organs. The usage of nanoparticles as a carrier for the delivery of payloads has passed preclinical studies and, in some diseases, has reached the clinical trials. Ordikhani *et al*.^12^ and Caron *et al*.^13^ used polymeric nanoparticles for targeted delivery of different therapeutic agents and neurotrophic factors for treating spinal cord injury sites. Porous nanomaterials emerged as a promising approach for biomedical applications due to the high specific surface area that leads to improved efficacy in the relevant application. Amongst various porous materials, silica nanoparticles with hydrophilic surface property, stability, biodegradability, low toxicity, photoluminescence properties, good biocompatibility, controllable size and easy surface modification will be an excellent candidate for drug delivery applications.^14,15^ So, we developed porous silica nanoparticle (PSi-NP)–loaded drugs armed with a targeting system to accumulate them in the lesion sites to overcome the challenges of traditional drug delivery systems.

One of the targeted drug delivery approaches that have been considered in recent years is the use of peptides that specifically identify the sites of injury and can carry the therapeutic and diagnostic agents to the site of injuries.^16,17^ Mann *et al*. introduced CAQK peptide (sequence: cysteine–alanine–glutamine–lysine) that targeted the proteoglycan complex in the traumatic brain injuries of animal models. Their results showed that CAQK specifically binds to the sites of the injuries in both mouse and human brain samples.^18^ Moreover, other studies showed that CAQK peptide targeted the lesion sites in animal models of spinal cord injury.^19-21^

In this study, we showed for the first time that the CAQK peptide could detect demyelinated areas in animal-demyelinated lesions. Therefore, we attempted to provide a multifunctional system using nanoparticles coated by the CAQK peptide to identify demyelinating lesions in relevant diseases. As a proof of concept, we were able to target MP to the glial scars in a demyelination animal model of multiple sclerosis in mice that reduced the inflammation.

## Materials and methods

### Animals

We purchased 8-week-old (bodyweight 21–25 g) C57BL/6 male mice from the Pasteur Institute (Karaj, Iran). The animals (42 mice in total) were kept in a temperature-controlled (23 ± 2°C) animal house under a 12-h light/dark period and randomly allocated to the animal groups. Food and water were available *ad libitum*. All experiments were performed in accordance with the International Guidelines for the Care and Use of Laboratory Animals. The Committee for Ethics in Animal Research at Tarbiat Modares University approved the experimental procedures (approval number: D52/6725). We tried to decrease the number of animals used during the study and their suffering. Mice were kept in a housing facility at least for 1 week to acclimatize before the experimental procedure.

### LPC-induced demyelination

To induce demyelination, lysolecithin [lysophosphatidylcholine (LPC), Sigma, St. Louis, USA] was injected into the corpus callosum (CC) to produce an animal model of demyelinated lesion as previously mentioned,^22^ using stereotaxic coordinates. Briefly, animals were anaesthetized with intraperitoneal injection of ketamine (70 mg/kg; Alfasan, Woerden, the Netherlands) and xylazine hydrochloride (10 mg/kg; Alfasan). After deep anaesthesia, mice were placed in a stereotaxic frame (Stoelting, Wood Dale, IL, USA) and the skull was exposed. Next, a small hole was made at the appropriate place based on the coordination obtained from the mouse stereotaxic atlas.^23^ One microliter of LPC 1% in phosphate-buffered saline (PBS) was slowly injected into the CC within 5 min, using a Hamilton syringe, and the needle was left in the place for an additional 10 min preventing LPC drawn up the injection track.

The extent of demyelination following LPC injection varies in frontal and caudal part of CC. A more focal demyelination in the rostral part^24^ and a distributed demyelination in caudal part^16^ have been observed. To check the lesion targeting by CAQK, LPC was injected into the frontal part of CC to have a focal demyelination and see the localization of peptide. To check the anti-inflammatory effect of MP, demyelination was induced in more caudal part to achieve more extensive inflammation with astrogliosis and microgliosis. Accordingly, the injected site coordinates were AP: +1 from bregma, L: 1.2, DV: 2.2, for frontal CC; and AP: −1.1 from bregma, L: 0.6, DV: 1.6, for caudal CC.

### Synthesis and modification of PSi-NPs

PSi-NPs were prepared by electrochemical etching of silicon according to our previous experiment.^25^ Briefly, a monocrystalline p-type silicon wafer [phosphorus-doped, 0.5 Ω cm, (100) oriented, Silicon Quest Inc. USA] was etched electrochemically to prepare PSi. For this purpose, 5-mAcm^−2^ current was applied in a mixture of aqueous hydrofluoric acid/ethanol for 5 min.

Then, a 250-mAcm^−2^ current was applied for 30–40 s to remove the porous layer. After that, the sample was sonicated overnight to fracture the free-standing PSi film and filtered through a membrane with a 0.22-µm pore size.

### Peptide synthesis and labelling

The peptides were synthesized and fluorescein amidites (FAM) labelled by ChinaPeptides Co., Ltd. with ordered sequences (FAM-CGGK and FAM-CAQK) with >96% purity. CGGK is a control peptide of the same length and overall charge as CAQK.^18^ Green FAM label was placed at the N-terminal of the peptides. The FAM-labelled peptides were lyophilized and stored at −20°C.

### Peptide conjugation to PSi-NPs

In order to modify the surface chemistry of the produced nanoparticles, 10-mg PSi-NPs dispersed in ethanol were mixed with 40 µl of 3-(ethoxymethyl)-propylamine silane. After stirring at room temperature overnight, the nanoparticles with amine-terminals were thoroughly washed with ethanol. Then, the particles were reacted with 1 ml of succinimidyl carboxymethyl ester-polyethylene glycol-maleimide at the concentration of 10 mg/ml in ethanol for 2 h at room temperature. The product was separated and washed with ethanol and deionized water. The surface-modified PSi-NPs were mixed with 500-µl aqueous solution of 1-mg/ml peptide, stirred for 2 h at room temperature and washed three times with water. The concentration of peptide solution was measured before and after introducing it to PSi-NPs to estimate peptide conjugation, which was approximated to be ∼45 nmol/mg (peptide/PSi-NPs).

### Drug loading

To prove that the targeting system works, we tried to load and deliver MP, a well-known anti-inflammatory drug, to the lesion site. Ten-milligram peptide-functionalized PSi-NPs were mixed with a solution of 15-mg/ml MP (as MP hemisuccinate) prepared in distilled water. The suspension was stirred at room temperature for 24 h. The drug-loaded nanoparticles were separated from the supernatant by centrifugation at 8000 *g* for 3 min and dispersed into PBS for further analysis. The ultraviolet–visible absorbance of MP at 247 nm was used to determine the drug loading by measuring the difference between the initial drug concentration and the concentration of the drug in the supernatant.

### Drug release studies

The drug release experiment was performed using dialysis bags with a 10-kDa weight cut-off. The dialysis bags were pre-treated using hot water at 70°C for 40 min. The drug-loaded PSi-NPs were poured into the dialysis bag with 1 ml of release media. Then, the dialysis bag was put into a glass bottle with 50-ml PBS. The solution was shaken at 100 rpm and 37°C. The concentration of the released drug was determined every day using the high performance liquid chromatography method according to the absorbance of the drug at 247 nm.

### Evaluation of peptide binding on tissue section

Using FAM-labelled CAQK peptide, the *ex vivo* experiments were run to evaluate the binding of the peptide on frozen brain sections of the demyelinated model. FAM-CGGK peptide was used as non-specific targeting control. The sections were incubated with the peptide dispersed in PBS-T with a concentration of 100 nM overnight at 4°C. Then, fluorescence imaging was done to detect FAM-labelled peptides on demyelinated sections.

### *In vivo* imaging

Mice were intravenously injected with FAM-labelled peptides (FAM-CAQK and FAM-CGGK) 6 days after lysolecithin-induced demyelination. After 2 h of circulation, the animals (three mice/group) were deeply anaesthetized with intraperitoneal injection of ketamine (70 mg/kg) and xylazine hydrochloride (10 mg/kg) and were placed in Bruker *In Vivo* Imager, and images were collected with 470-nm excitation wavelength, 535-nm emission wavelength and 3-s exposure time. Immediately, the mice were euthanized, and brain tissue was obtained.

### Animal groups in MP targeting experiment

Mice were randomly divided into four groups each including six mice: (i) intact: non-LPC injected and non-treated mice; (ii) LPC: LPC injected and non-treated mice; (iii) MP: LPC injected and MP-treated mice (0.24 mg/kg); and (iv) MP@CAQK-PSi: LPC injected and treated with MP-loaded CAQK-modified PSi (MP dose was equal to 0.24 mg/kg). Treatments were delivered intravenously started on Day 5 post-LPC injection. In each group, three mice were sacrificed 24 h after the treatment (Day 6 post-LPC), and three mice were sacrificed after 48 h (Day 7 post-LPC).

### Immunostaining and histological investigations

The animals were deeply re-anaesthetized using ketamine and xylazine and transcardially perfused and post-fixed in PFA for 24 h as previously mentioned. The next day, we immersed the tissues in 30% sucrose for 24–48 h, and the brains were embedded in optimum cutting temperature (Bio-Optica, Italy). Coronal sections (8-μm thick) were prepared by using a cryostat instrument (Histo-Line Laboratories, Milan, Italy).

After that, the permeability and blocking were done respectively by 0.2% Triton X-100 and 10% normal goat serum for 1 h. Then, the sections were incubated with primary antibodies against myelin basic protein (MBP) as a marker expressed in myelinating oligodendrocytes, glial fibrillary acidic protein (GFAP) as a marker of astrocytes and ionized calcium-binding adaptor molecule 1 (Iba1) as a marker of microglia. Incubation was done for overnight at 4°C followed by three times of washing with PBS and 1-h incubation with appropriate secondary antibodies. A list of primary and secondary antibodies used in this study is presented in Table 1. Next, the sections were washed again, and 4,6-diamidino-2-phenylindole (DAPI) was used to stain the nucleus. The intensity of immunoreactivity for each staining was measured by ImageJ software as previously reported.^26^ For each animal, six sections were quantified and then averaged and entered into mean calculation for three mice per group. We captured the images using Olympus BX41 fluorescence microscopy for further analysis.

**Table 1:** List of primary and secondary antibodies used in this study

Antibody	Species isotype	Company	Dilution	Label
GFAP	Chicken polyclonal	Aves	1:2000	-
MBP	Chicken polyclonal	Aves	1:1000	-
Iba1	Rabbit polyclonal	Wako, 019-19741	1:1000	-
Rabbit IgG	Goat anti-rabbit	Thermo Fisher, A11036	1:1000	Alexa Fluor® 568
Chicken IgY	Rabbit anti-chicken	Abcam, ab6751	1:500	Texas Red

GFAP, glial fibrillary acidic protein; Iba1, ionized calcium-binding adaptor molecule; MBP, myelin basic protein.

Haematoxylin and eosin staining was also applied for evaluating the severity of inflammation based on a grading scale as follows: no infiltration, 0; mild cellular infiltration to CC, 1; moderate infiltration, 2; severe infiltration, 3; massive infiltration, 4.^27,28^Supplementary Fig. 2 shows sample tissue sections for each observed score. Six sections per animal and three mice per group were used for quantification. Olympus BX41 fluorescence microscopy was used for capturing the images.

### Statistical analysis

The results of the present study were analysed using GraphPad Prism software (version 6.1). Histological data were measured using one-way ANOVA followed by Tukey *post hoc* test. All the results were expressed as mean ± SEM and *P*-values of <0.05 were considered statistically significant. The scientist who performed the experiment was not blinded to the sample.

## Results

### Lysolecithin-induced demyelination model and CAQK binding to demyelination area

To evaluate the binding of CAQK peptide to demyelinating lesions, demyelination was induced by direct injection of lysolecithin 1% (1 µl) into the CC of mice. In this well-documented model,^24^ following lysolecithin injection–defined areas of demyelination was induced in the white matter.

After 6 days, a group of the mice was sacrificed and their brains were collected, post-fixed and stained against myelin using FluoroMyelin (Supplementary Fig. 1A). To characterize the binding of FAM-labelled peptides to the demyelinated brain area, FAM-labelled peptides were added on PBS equilibrated mouse brain sections. When the sections were compared between control peptide (CGGK) and CAQK, we observed that CGGK was absent, while CAQK was observed at the site of demyelination (Supplementary Fig. 1B).

**Figure 1:**
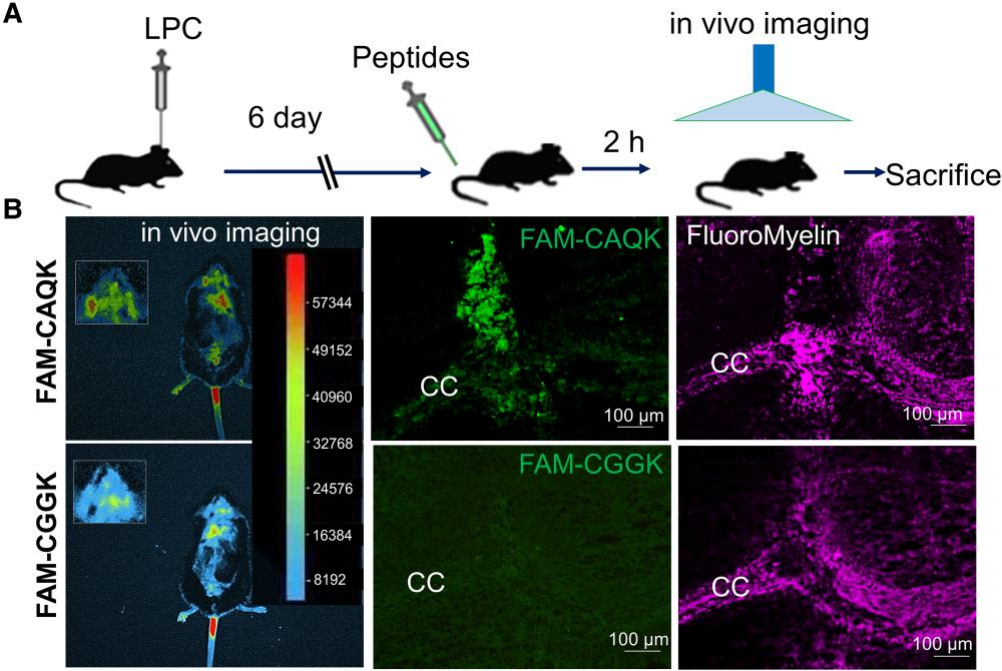
**CAQK binding to demyelinated area**. (**A**) Timeline of animal modelling, peptide injection, *in vivo* imaging and brain sampling. (**B**) *In vivo* imaging of mice intravenously injected with FAM-labelled peptides 6 days after LPC-induced demyelination and 2 h after peptide injection. Values in the heat map range from 8192 to 57 344. Middle panel shows CAQK peptide binding to the demyelination areas of the brain. FluoroMyelin staining of mice brain sections showed the presence of a demyelinated area within the CC (*n* = 6). Scale bar: 100 μm. CAQK, targeting peptide; CC, corpus callosum; CGGK, control peptide; FAM, fluorescein amidites; LPC, lysophosphatidylcholine.

We used *in vivo* imaging approach to track the binding of peptides to the demyelinated area. Therefore, mice were intravenously injected with FAM-labelled peptides 6 days after induction of demyelination. After a 2-h circulation time, the images showed that CAQK bound to the demyelination area of the brain (Fig. 1A and B). Shortly after *in vivo* imaging, mice were perfused, and their brains were sectioned. The sections were imaged with fluorescent microscopy by looking at intrinsic emission from the FAM tag on the peptides; also, staining was done using FluoroMyelin. The results showed that CAQK binding was specific to the sites of demyelination, compared with the control peptide (Fig. 1B).

### CAQK co-localized with glial cells in demyelination areas

**Figure 2:**
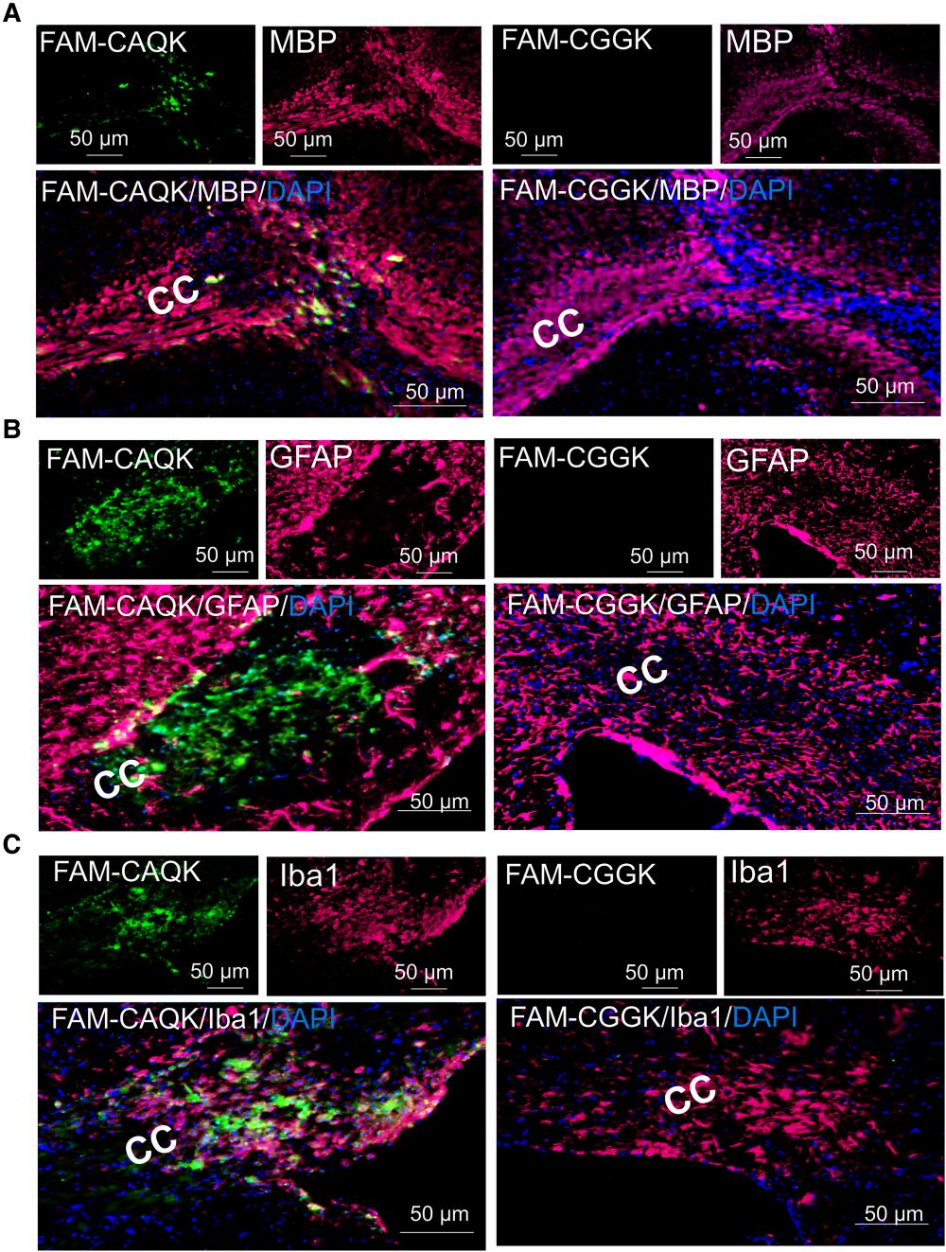
**Co-immunofluorescence of FAM-labelled peptides with glial cells in demyelinated area**. Frozen sections from the CC region of LPC-demyelinated mice intravenously injected with FAM-CAQK or FAM-CGGK (green) co-stained for MBP (**A**), GFAP (**B**) or Iba-1 (**C**), all co-stained with DAPI. *n* = 3 mice per experimental group. Scale bar: 50 μm. CAQK, targeting peptide; CC, corpus callosum; CGGK, control peptide; DAPI, 4,6-diamidino-2-phenylindole; FAM, fluorescein amidites; GFAP, glial fibrillary acidic protein; Iba1, ionized calcium-binding adaptor molecule 1; MBP, myelin basic protein.

To identify the particular cellular targets of CAQK in the demyelinated tissue from the mouse brain, 6 days after induced demyelination, the mice were intravenously injected with 50 nmol of FAM peptides dissolved in PBS. After 2 h, animals were sacrificed and perfused intracardially with PBS. Next, their brains were collected, post-fixed and stained against MBP, GFAP and Iba1. The results showed that MBP was decreased (Fig. 2A), while GFAP and Iba1 expression was increased in demyelinated areas (Fig. 2B and C). It was notable that GFAP was observed mostly at the edge of the lesion. The signal from intravenously injected CAQK was co-localized with demyelination and Iba1^+^ cells (Fig. 2A and C), whereas CAQK did not co-localize with astrocyte cells in demyelination sites (Fig. 2B). In the control CGGK group, no fluorescence signal was observed at the demyelination site (Fig. 2A–C).

### Nanoparticle size, drug loading and drug release

**Figure 3:**
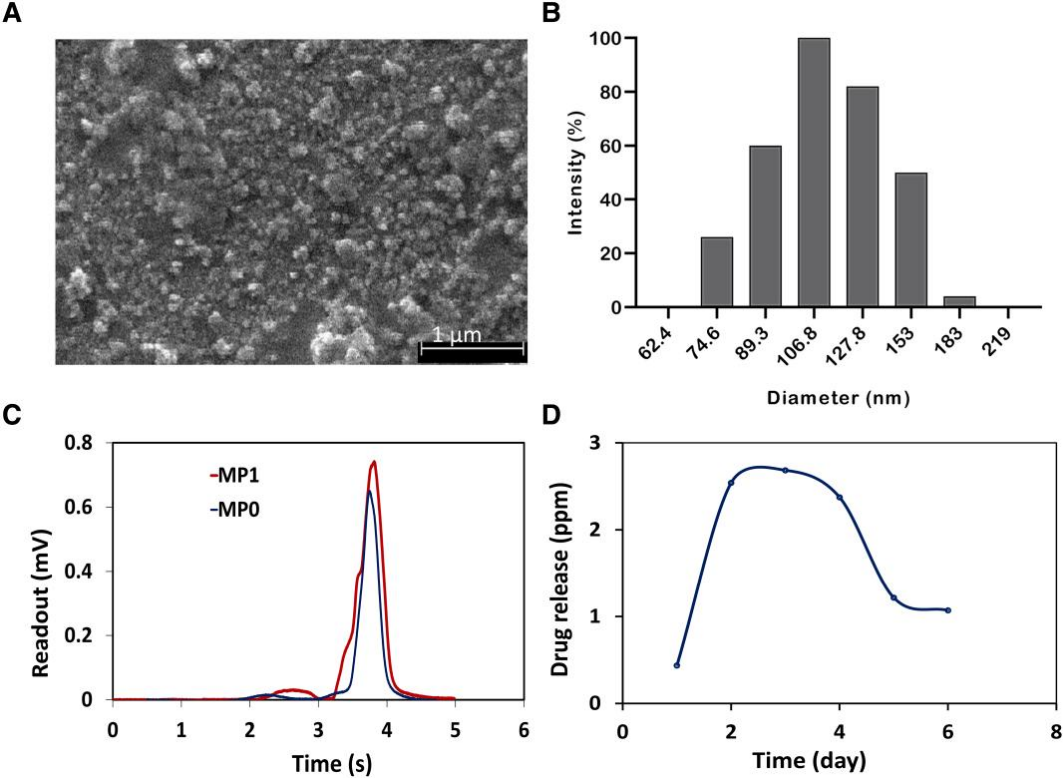
**Characterization of porous silicon nanoparticles (PSi-NPs)**. (**A**) SEM image of PSi-NPs. (**B**) Dynamic light scattering particle size distribution profiles of PSi-NPs at room temperature with an etching time of 5 min and current density 5 mA/cm^−2^. (**C**) Drug concentration for MP, MP0 and MP1 was measured for drug loading calculation. (**D**) *In vitro* release of MP from PSi-NPs. MP0, initial MP concentration; MP1, MP concentration in supernatant after drug loading; SEM, scanning electron microscopy.

The produced nanoparticles were measured for size, drug loading and drug release. The mean size of the obtained PSi-NPs, measured by transmission electron microscopy and dynamic light scattering, was about 106 nm [Fig. 3A (TEM) and Fig. 3B (dynamic light scattering)]. The ultraviolet–visible absorbance of MP at 247 nm was measured to determine the drug loading by measuring the difference between the initial drug concentration and the concentration of the drug in the supernatant after removing the nanoparticles (Fig. 3C). The amount of drug loading into the nanoparticles was estimated to be 0.8 mg/mg (MP/PSi-NPs), equal to 53 wt% of the total drug was used for loading onto the nanoparticles.

**Figure 4:**
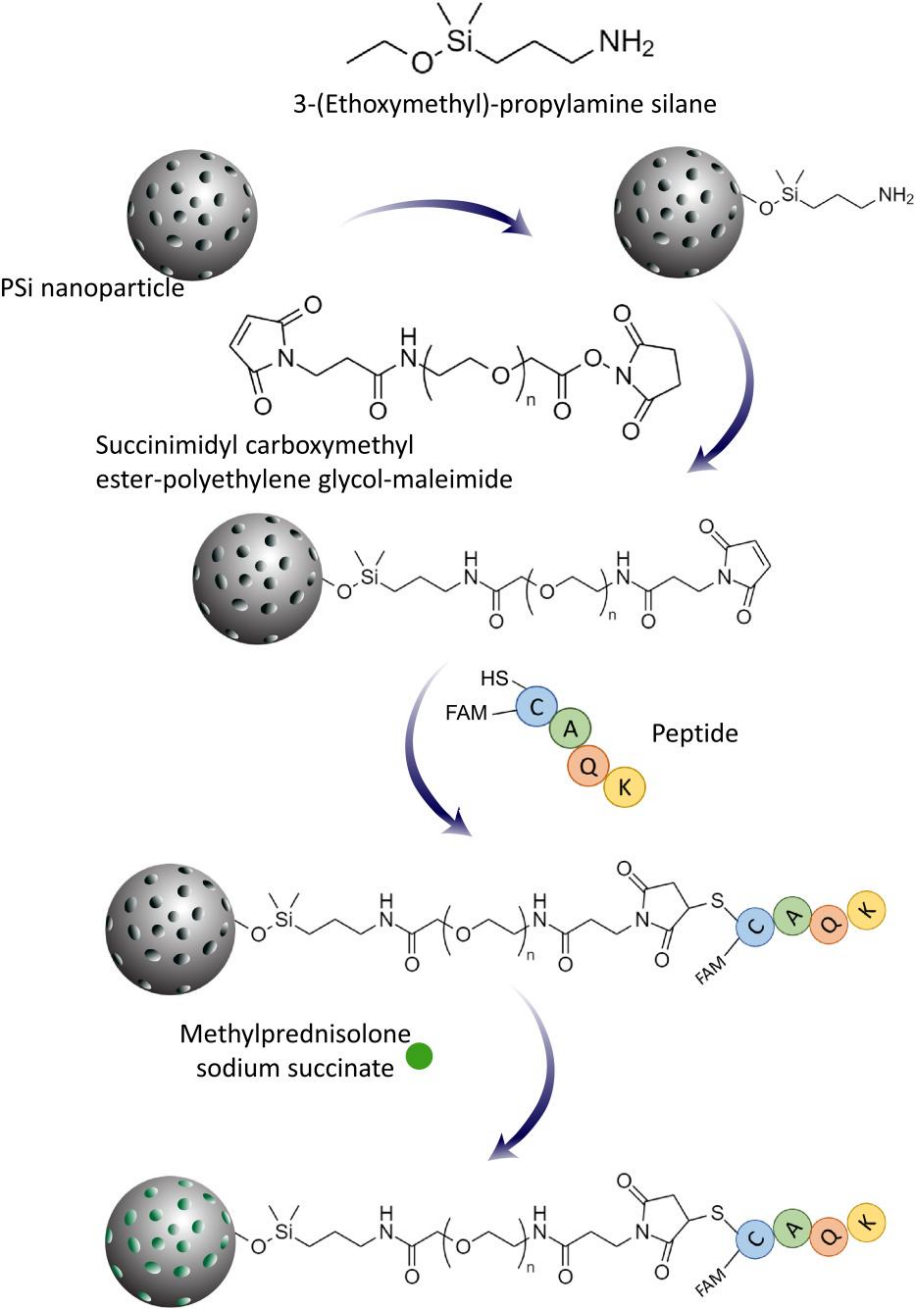
**A schematic illustration for the surface modification and drug loading of the silicon nanoparticle**. PSi-NPs dispersed in ethanol were mixed with 3-(ethoxymethyl)-propylamine. Then, the particles were reacted with succinimidyl carboxymethyl ester-polyethylene glycol-maleimide prepared in ethanol at room temperature. The surface-modified PSi-NPs were mixed with aqueous solution of peptide. For drug loading, peptide-functionalized PSi-NPs were mixed with a solution of MP hemisuccinate prepared in distilled water and stirred at room temperature for 24 h. SH, SH functional group on cysteine residue.

Since the *in vitro* drug release is an important issue in drug development and quality control, we investigated the MP release profile from PSi-NPs at 37°C (Fig. 3D). The release value was increased to 2.63 ppm in the first 3 days and then decreased to 1.02 ppm till Day 6. This decrease seems to be due to the drug decomposition. In a study by Trissel *et al*.,^29^ the stability of MP was examined, showing 8% loss after 2 days and about 13% loss after 3 days at 23°C and 10% loss after 30 days at 4°C, which revealed some decomposition of the drug in the samples by the time and the incubation temperature. In this study, incubation was performed at 37°C, and thus a higher rate of decomposition was expected that led to more than 61% decomposition. It means that in our setting, the drug decomposition after Day 3 surpassed the drug release and therefore the maximum drug entity was observed on Day 3. The surface of silicon nanoparticles was modified with 3-(ethoxymethyl)-propylamine saline followed by conjugation of succinimidyl carboxymethyl ester-polyethylene glycol-maleimide to facilitate peptide binding. A further modification was done by conjugation of the CAQK peptide to the linker. Next, the MP molecules were loaded on the silicone pores (Fig. 4).

### CAQK-PSi binding was specific to the demyelination area

**Figure 5:**
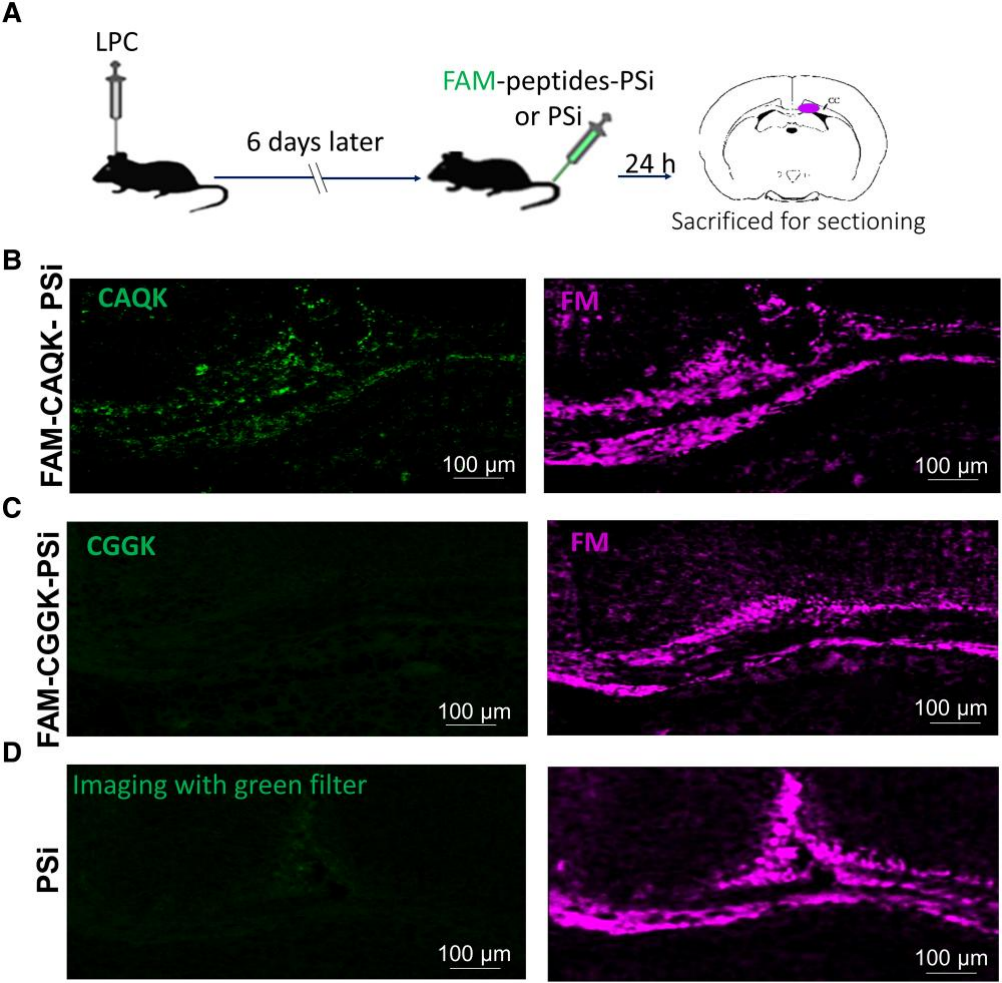
**CAQK-PSi targeted the demyelination area**. (**A**) Timeline of animal modelling and intravenously injection of PSi, PSi modified by FAM-labelled peptides followed by brain slice preparation. (**B**–**D**) CAQK-PSi binding was specific to demyelination sites (**B**), whereas low bindings of the control peptide-PSi (CGGK-PSi) (**C**) and PSi (**D**) were observed. FluoroMyelin (FM) staining of mice brain sections was used to show the demyelinated areas within the CC. *n* = 3 mice per experimental group. Scale bar: 100 μm. CAQK, targeting peptide; CC, corpus callosum; CGGK, control peptide; FAM, fluorescein amidites; LPC, lysophosphatidylcholine.

To characterize the stability of binding of CAQK to the demyelinated brain area after binding to PSi and to verify the peptide capability to target PSi to the lesion site, CAQK-PSi was injected into animals with LPC-induced demyelination. Six days after induction of demyelination (a time point known as maximum demyelination following LPC), nanoparticles attached with FAM-labelled peptides (CAQK-PSi and CGGK-PSi) or without peptides (PSi) were intravenously injected into the mice (Fig. 5A). Our results showed that similar to the binding pattern of FAM-CAQK on demyelinated brain sections obtained from demyelinated model animals, CAQK-PSi binding was specific to demyelination sites (Fig. 5B), whereas the binding of control PSi (CGGK-PSi) and PSi was minor (Fig. 5C and D).

### CAQK-PSi targeted drug delivery to the demyelination area

**Figure 6:**
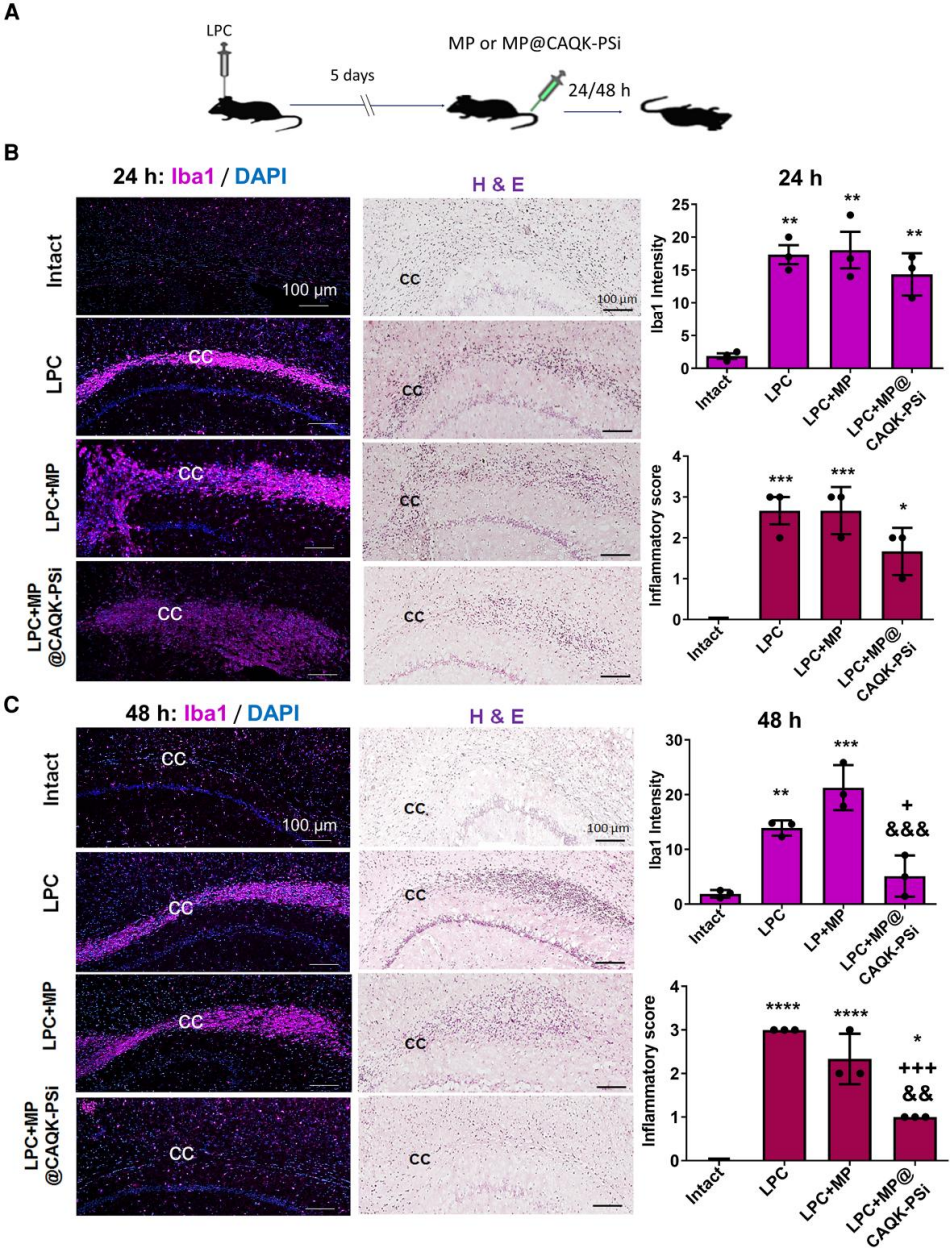
**Effect of MP-loaded porous silicon nanoparticles (PSi-NPs) on the inflammation indices in lesion area**. (**A**) Timeline of animal modelling, intravenously injection of PBS, MP or MP-loaded PSi-NPs (MP@CAQK-PSi) and brain slice preparation. (**B, C**) Immunostaining against Iba1 as a microglial marker in lesion regions of the CC along with haematoxylin and eosin (H&E) staining for evaluation of inflammatory cell infiltration in lesion regions of the CC, respectively at 24 h (**B**) or 48 h (**C**) after treatment. Quantification results of immunostaining against Iba1 and H&E staining mentioned in **B** and **C** are presented in right panel graphs. *P < 0.05, **P < 0.01, ***P < 0.001 and ****P < 0.0001 compared with Intact group, +*P* < 0.05 and +++*P* < 0.001 compared with LPC group and &&*P* < 0.01 and &&&*P* < 0.001 compared with free MP (LPC + MP) group as evaluated by one-way ANOVA followed by Tukey *post hoc* test. *n* = 3 mice per experimental group (six sections were quantified for each mouse). Scale bar: 100 μm. CAQK, targeting peptide; CC, corpus callosum; Iba1, ionized calcium-binding adaptor molecule 1; LPC, lysophosphatidylcholine.

To test whether CAQK-PSi could specifically target the loaded drug to the lesion site following LPC-induced demyelination, MP, a well-known anti-inflammatory drug, was loaded into the PSi and injected intravenously to evaluate possible inhibition of the inflammation process in the lysolecithin-injected site (Fig. 6A). The efficacy of loaded and targeted MP was compared with free MP injected at the same dose. Comprehensive immunofluorescence and pathological evaluations were performed in four animal groups (Intact, LPC, LPC + MP and LPC + MP@CAQK-PSi) in order to evaluate the possible reduction in microglial activation.

Analysis of immunostaining data indicated that in comparison with the intact group, injection of LPC significantly increased the intensity of Iba1 staining at Days 6 (*P* < 0.01) and 7 (*P* < 0.001) (Fig. 6B and C). Furthermore, application of free MP (LPC + MP group) and MP loaded at PSi-NPs (LPC + MP@CAQK-PSi) at a single dose of 0.24 mg showed that the MP@CAQK-PSi significantly reduced the levels of microglial activation in the lesion site in the CC, compared with the LPC (*P* < 0.05) and LPC + MP (*P* < 0.001) groups as evaluated after 48 h (Fig. 6C). In addition, there were no significant differences between the three mentioned groups on 24 h after treatment (Fig. 6B and C).

The pathological analysis showed an increased infiltration of inflammatory cells throughout the CC in LPC-treated animals as compared with the intact group on Days 6 (*P* < 0.001) and 7 (*P* < 0.0001) post-LPC injection (Fig. 6B and C). However, there was a significant difference between LPC + MP@CAQK-PSi and LPC groups (*P* < 0.001) and LPC + MP@CAQK-PSi and LPC + MP groups (*P* < 0.01) at 48 h after treatment. Therefore, these data showed that diffuse severe infiltration of inflammatory cells throughout the CC was decreased in MP-loaded CAQK-PSi-treated mice after 48 h (Fig. 6C).

### MP-loaded PSi-NPs ameliorated CC astrocyte activation following LPC demyelination

**Figure 7:**
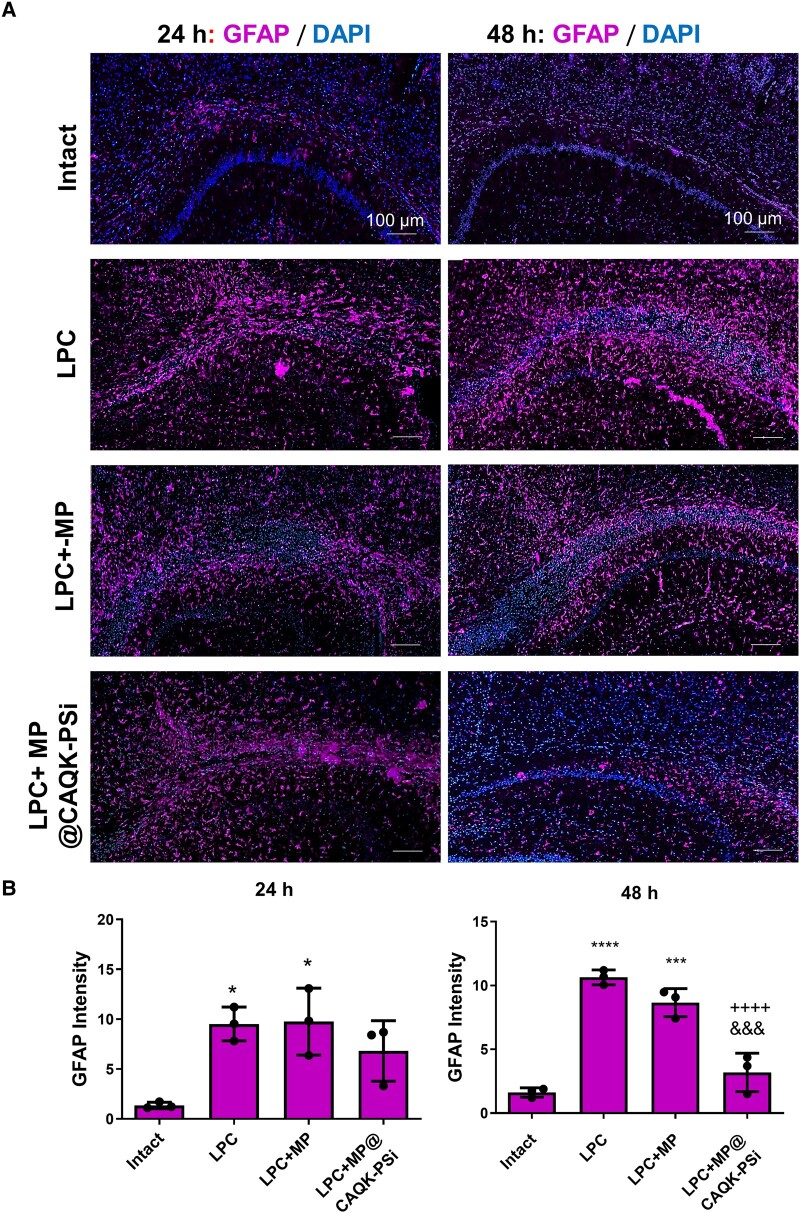
**Effect of MP-loaded porous silicon nanoparticles (MP@CAQK-PSi) on astrocyte reactivation in lesion areas**. (**A**) Immunostaining against GFAP (red) as astrocyte marker in the lesion region. DAPI (blue): nuclei stain. (**B**) Quantification of immunostaining data. *P < 0.05, ***P < 0.001 and ****P < 0.0001 compared with Intact group, ++++*P* < 0.0001 compared with LPC group and &&&*P* < 0.001 compared to free MP (LPC + MP) as evaluated by one-way ANOVA followed by Tukey *post hoc* test. *n* = 3 per experimental group (six sections were quantified for each mouse). Scale bar: 100 μm. CAQK, targeting peptide; DAPI, 4,6-diamidino-2-phenylindole; FAM, fluorescein amidites; GFAP, glial fibrillary acidic protein; LPC, lysophosphatidylcholine.

In order to evaluate the effect of MP or MP nanoformulation on glial activation, the level of astrocyte activation was assessed using immunostaining against GFAP as a reactive astrocyte marker (Fig. 7). Analysis of immunostaining data indicated that the intensity of GFAP fluorescent signals was increased on Days 6 and 7 after the induced demyelination model using lysolecithin compared with the intact group (Fig. 7A and B). In addition, quantitative analysis showed that free MP (LPC + MP group) and MP-loaded porous silicon nanoparticles (PSi-NPs) (LPC + MP@CAQK-PSi group) did not decrease glial activation at 24 h after the treatment (Fig. 7A and B).

The intensity of GFAP fluorescent signals was significantly decreased in mice treated with MP-loaded PSi-NPs (LPC + MP@CAQK-PSi) as compared with the LPC (*P* < 0.0001) and free MP (LPC + MP) (*P* < 0.001) groups at 48 h after the treatment (Fig. 7A and B).

## Discussion

One of the main promising aspects of nanoparticle-based drug delivery is the possibility of targeted delivery.^16^ In recent years, novel therapeutic approaches have been investigated for the development of safe drug delivery in the context of neurological disorders. In 2016, Mann *et al*.^18^ introduced a peptide with four amino acids that selectively recognized the brain injuries and accumulated at the site of injury. Here, we investigated whether CAQK recognizes demyelinated lesions in the brain sections obtained from animal models of demyelination. Moreover, we demonstrated that this peptide alone (FAM-CAQK) or together with silicon nanoparticles, accumulated in the injured areas when being injected intravenously into the mice model with myelin insult. The nanocarrier system loaded with MP reduced the levels of inflammatory factors and astrocyte reactivation, which was induced by LPC injection. LPC-induced demyelination is a well-established model of focal demyelination for the evaluation of the processes of inflammatory demyelination and myelin repair. The toxin-based model of LPC-induced demyelination could be enhanced by manipulating inflammatory response.^28,30^ Our results showed that local LPC administration into the white matter (mice CC) increased reactivated astrocytes and microglia; besides, it led to severe infiltration of inflammatory cells around or inside the legion area (Figs 6 and 7).

The results of *in vivo* live imaging indicated that FAM-CAQK reached the demyelinated lesions when being injected intravenously (Fig. 1B). Furthermore, our histological results showed that FAM-CAQK was accumulated around the glial cell population, including astrocytes and microglia (Fig. 2A–C). In an available study by Mann *et al*.,^18^ the authors reported that peptide bounded not often to Olig2- and NG2-positive cells. In a recent report by Abi-Ghanem *et al*., CAQK peptides targeted the demyelination sites in EAE, LPC and cuprizone models of multiple sclerosis. They suggest that CAQK peptides are associated with tenascins.^31^ Additionally, in whole-body imaging, we observed some off-target accumulation of CAQK peptide. Peptide detection in some organs with high level of blood circulation of those with a role in peptide metabolism like as liver, kidney and lung is expectable. For CAQK peptide, its off-target accumulation was previously reported in the kidney.^18^ While the use of peptides as drug carriers may exert important undesirable side effects,^32^ Mann *et al*.^18^ did not observe evidence of potential side effects or limitations in the use of CAQK peptide coming from off-target areas.

In our study, we used PSi-NPs as the drug carrier with the approximate size of 106 nm (Fig. 3A and B), which was in the range of particle sizes that penetrated the blood–brain barrier. The results of the drug release rate showed that the amount of MP from PSi competed with the drug loss showed a maximum of drug entity on Day 3 (Fig. 3D).

MP is frequently used as a treatment for relapsing–remitting attacks in both oral and intravenously high doses for 3–5 days.^33,34^ The pathologic hallmark of multiple sclerosis is the formation of focal demyelinated plaques within the CNS, characterized by a variable degree of astrogliosis, inflammation and degenerative/reparative processes. They might be numerously formed in white matter and the grey matter areas.^35,36^ Thus, studies have shown that using corticosteroid drugs may be promising by their anti-inflammatory effects.^37^

In the current study, for proving the concept that our designed nanodelivery system assists us with avoiding systemic side effects of a high dose of MP, we loaded MP into the nanosystem coated with FAM-labelled CAQK peptide to target the brain demyelination sites in order to reduce the ongoing inflammation. The histopathology results and immunostaining data indicated that the application of MP-loaded PSi-NPs (MP@PSi-CAQK) at a single dose of 0.24 mg significantly reduced the levels of microglial activation in the lesion induced in the CC by LPC injection, when compared with the LPC and free MP (LPC + MP) groups, 48 h after intravenous injection (Fig. 6C). Over-activation of microglia and the NLRP3 inflammasome have been explained in the pathogenesis of multiple sclerosis. Both experimental data and the post-mortem and clinical studies have shown an increased expression of NLRP3 inflammasome complex in microglial and other immune cells.^38^ The mechanism of action of MP on microglial cells is not fully understood, but some studies suggest that it may inhibit microglial activation and promote an anti-inflammatory phenotype. MP is widely used to treat acute relapses of multiple sclerosis. MP pulse therapy caused a marked shift towards an anti-inflammatory monocyte phenotype as revealed by an altered gene expression profile.^39^ In a mice model of Alzheimer’s disease, MP inhibited the activation of microglia in the cortex and hippocampus.^40^ In mice with spinal cord injury, MP inhibited microglial activation and suppressed A1 neurotoxic type of reactive astrocytes.^41^ These reports support our finding about the effect of targeted MP on microglia and may explain some possible mechanisms for its action in the demyelination context.

Furthermore, as shown in Fig. 7A and B, the level of GFAP expression was increased after LPC induction of demyelination, which confirmed the activation of astrocytes in the damaged area.^42^ Following CNS injuries such as trauma, stroke, infection, autoimmune responses or degenerative disease,^43-45^ astrocytes initially proliferate and then migrate towards the damage site and form a very tight glial scar to keep normal cells apart from inflammatory cell infiltration.^46,47^ We analysed the level of astrocytes activation, and our findings showed that intravenous injection of MP-loaded PSi-NPs (MP@PSi-CAQK) significantly alleviated the intensity of GFAP expression following LPC injection (Fig. 7). In agreement with our results, a previous study by Liu *et al*.^48^ also demonstrated that MP significantly downregulated the expression of GFAP, neurocan, chondroitin sulphate proteoglycans and phosphacan in an induced reactivated astrocytes model, when applied at concentrations of 10 and 50 µM. In addition, it has been shown that MP could decrease the expression of chondroitin sulphate proteoglycans in glial scars in the experimental model of cerebral ischemia.^49^ Intranasal delivery of MP to the brain reduced the expression of TNF-α, NO and GFAP, which implies for direct anti-inflammatory effects of MP in the neuroinflammatory conditions.^50^ MP inhibited interferon gamma and interleukin-17 expression and production by immune cells infiltrated into the CNC following experimental autoimmune encephalomyelitis.^51^ Additionally, Lee *et al*. showed that a high dose of free MP (100 mg/kg) and a low dose of MP loaded in nanoparticles (10 mg/kg) have the same effect in reducing astrocyte activation in the spinal cord-injured mice. Therefore, their results showed that the nanoparticle was able to reduce the amount of required drug for diminishing the inflammation.^52^ In another report, the inhibitory effect of MP on inflammatory cascade has been observed with a single dose of 30 or 60 mg/kg in the rat model of spinal cord injury.^27^ Consistent with our study, Sun *et al*. designed mesoporous silica nanoparticles modified with fluorescein isothiocyanate and CAQK peptide for targeted delivery of anti-inflammatory agents in the spinal cord injury model. Their result showed that CAQK-modified nanoparticles successfully delivered therapeutic agents into the site of spinal cord injury and inhibited the activation of astrocytes due to the reduction of interleukin-17 and interleukin-17-related inflammatory factors.^11^ Targeted drug delivery to reactive astrocytes in neurological disorders can be an approach to control the progress of the disease. For instance, some reports showed that Schwann cells can remyelinate axons in spinal cord lesions in the absence of GFAP.^53^ In another study, targeting the ephrine-A5 expression in astrocytes promoted recovery in the stroke model.^54^

Our findings suggest that in the pathophysiological process of demyelination, MP@PSi-CAQK may suppress astrocyte reactivation and inflammatory processes at a much lower dose than reported in previous studies. Therefore, targeted delivery of nanoparticles that are loaded with MP or other drugs via modification with CAQK may be useful for neuroprotection as well as enhancing endogenous repair and setting up a variety of delivery system into the human brain lesions.

## Conclusion

In the present study, CAQK peptide could efficiently bind to the demyelination lesion in mouse. When applied *in vivo*, CAQK delivered MP-loaded PSi-NPs to the lesion site. It seems that this PSi-NP-CAQK is a promising approach for targeted drug delivery to lesion areas, especially in patients with multiple sclerosis.

## Supplementary Material

fcad325_Supplementary_Data

## Data Availability

Data are presented in the main article and Supplementary material. Additional details are available upon formal request to the corresponding author.
