# Supplementary material for: Targeted drug delivery into glial scar using CAQK peptide in a mouse model of multiple sclerosis
Source: Brain Commun. 2023 Nov 27;5(6):fcad325. doi: 10.1093/braincomms/fcad325 (PMC10724044; doi:10.1093/braincomms/fcad325)
Supplement: fcad325_Supplementary_Data [file fcad325_supplementary_data.pdf]

Supplementary information for:

Targeted drug delivery into glial scar using CAQK peptide in a mouse model of multiple sclerosis

Leila Zare[1,2], Safoura Rezaei[3], Elaheh Esmaeili[1,2], Khosro Khajeh[3,4], Mohammad Javan[1,2,5]

1. Department of Physiology, Faculty of Medical Sciences, Tarbiat Modares University, Tehran, Iran.

2. Institute for Brain and Cognition, Tarbiat Modares University, Tehran, Iran.

3- Department of Nanobiotechnology, Faculty of Biological Sciences, Tarbiat Modares University, Tehran, Iran

4- Department of Biochemistry, Faculty of Biological Sciences, Tarbiat Modares University, Tehran, Iran.

5- International Collaboration on Repair Discoveries (ICORD), University of British Columbia, Vancouver, British Columbia, Canada

---

* Corresponding author: Mohammad Javan, P.O Box: 144115-331, Tehran. Iran.

Email: mjavan@modares.ac.ir

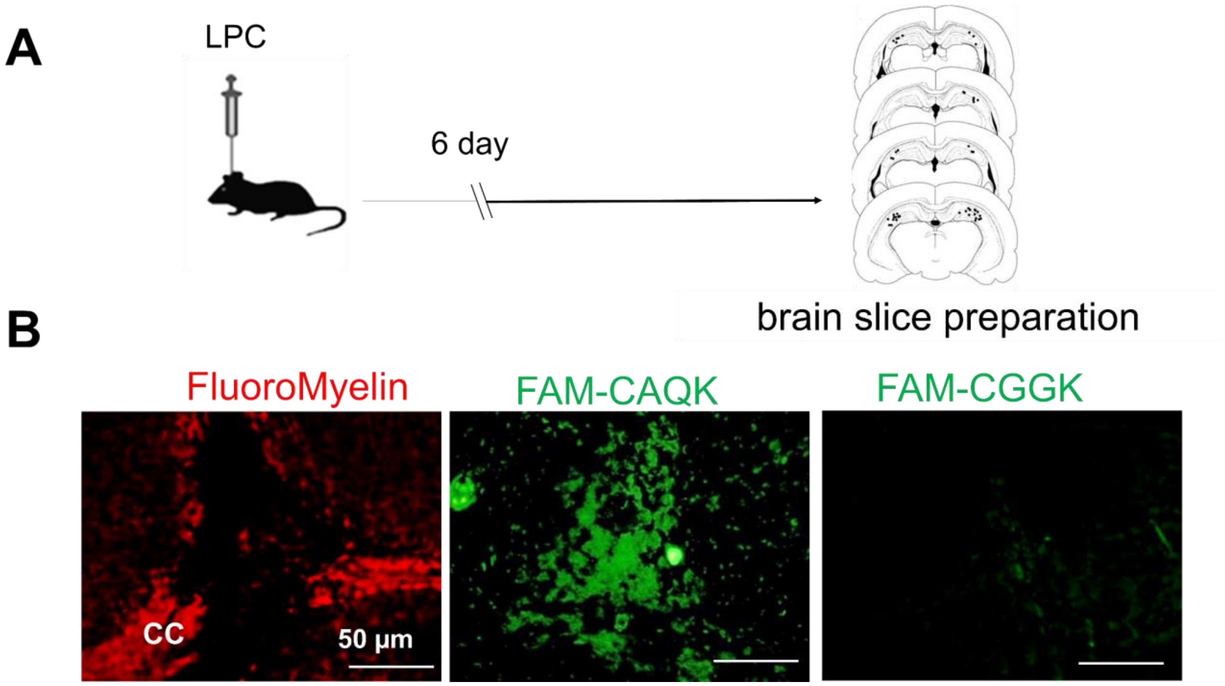

Supplementary figure 1: Mice brain sections obtained from LPC-induced demyelination model and CAQK binding to the demyelination area. A) Timeline of animal modeling and sacrifice for brain slice preparation. B) FluoroMyelin staining of mice brain sections showed the presence of a demyelinated area within the CC, along with fluorescence signal of FAM-CAQK and the lack of FAM-control peptide fluorescence in the mice brain section (n=6); LPC: lysophosphatidylcholine; CAQK: targeting peptide; CGGK: control peptide; FAM: fluorescein amidites.

Supplementary figure 2

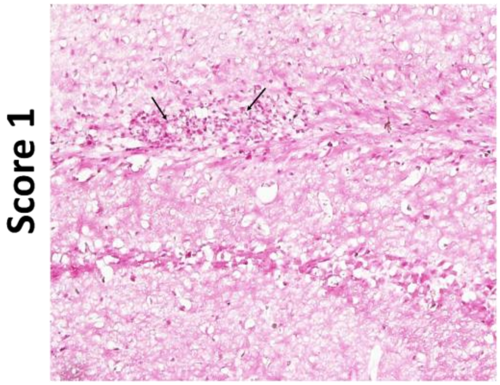

Shows focal mild infiltration of inflammatory cells throughout corpus callosum, mag:400X

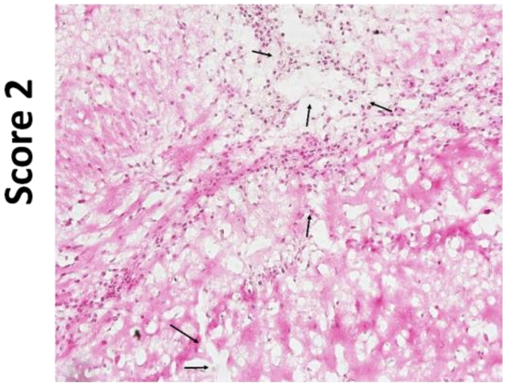

Shows diffused moderate infiltration of inflammatory cells throughout corpus callosum, mag:400X

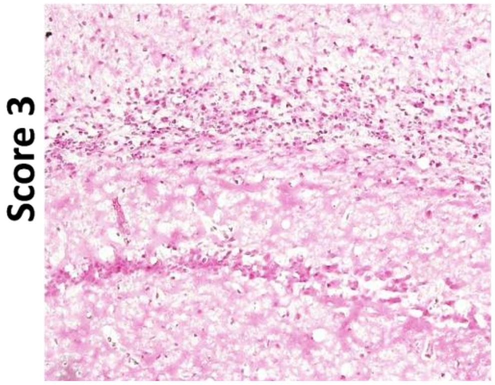

Shows diffuse severe infiltration of inflammatory cells throughout corpus callosum, mag:400X

Supplementary figure 2: Sample representative images for each score in H&E-stained slides presented in Fig. 4 B and C. Arrow heads show the relevant infiltrated cells.
